# Heart disease in the Netherlands: a quantitative update

**DOI:** 10.1007/s12471-013-0504-x

**Published:** 2013-12-17

**Authors:** M. J. G. Leening, S. Siregar, I. Vaartjes, M. L. Bots, M. I. M. Versteegh, R.-J. M. van Geuns, J. J. Koolen, J. W. Deckers

**Affiliations:** 1 Department of Cardiology, Erasmus MC – University Medical Center Rotterdam, P.O. Box 2040, 3000 CA Rotterdam, the Netherlands; 2 Department of Epidemiology, Erasmus MC – University Medical Center Rotterdam, Rotterdam, the Netherlands; 3 Department of Cardiothoracic Surgery, Leiden University Medical Center, Leiden, the Netherlands; 4 Department of Epidemiology, Julius Center for Health Sciences and Primary Care, University Medical Center Utrecht, Utrecht, the Netherlands; 5 Dutch Heart Foundation, The Hague, the Netherlands; 6 Supervisory Committee for Cardiac Interventions in the Netherlands, Utrecht, the Netherlands; 7 Department of Cardiology, Catharina Hospital, Eindhoven, the Netherlands; 8 National Cardiovascular Data Registry, Amsterdam, the Netherlands

**Keywords:** Cardiovascular disease, Mortality, Morbidity, Interventions, Registries, Statistics

## Abstract

In this review we discuss cardiovascular mortality, incidence and prevalence of heart disease, and cardiac interventions and surgery in the Netherlands. We combined most recently available data from various Dutch cardiovascular registries, Dutch Hospital Data (LMR), Statistics Netherlands (CBS), and population-based cohort studies, to provide a broad quantitative update. The absolute number of people dying from cardiovascular diseases is declining and cardiovascular conditions are no longer the leading cause of death in the Netherlands. However, a substantial burden of morbidity persists with 400,000 hospitalisations for cardiovascular disease involving over 80,000 cardiac interventions annually. In the Netherlands alone, an estimated 730,000 persons are currently diagnosed with coronary heart disease, 120,000 with heart failure, and 260,000 with atrial fibrillation. These numbers emphasise the continuous need for dedicated research on prevention, diagnosis, and treatment of heart disease in our country.

**Electronic supplementary material:**

The online version of this article (doi:10.1007/s12471-013-0504-x) contains supplementary material, which is available to authorized users.

## Introduction

This manuscript provides an update on the current number of persons with cardiovascular and cardiac disease manifestations in the Netherlands. Although the mortality from cardiovascular disease (CVD) in our country has declined, its disease burden remains high. Therefore, recent data on the number of patients with specific clinical cardiac disease entities, those having undergone surgical or percutaneous procedures, as well as estimates of the number of hospitalisations for cardiac reasons, are also given.

Data have been derived from various sources. Because it was not possible to obtain information from the year 2012 in every instance, exact present-day numbers of procedures or hospitalisations may be slightly dissimilar from those provided herein. Still, we hope that our numbers will provide some insight into the actual burden of heart disease in the Netherlands and its management.

## Cardiovascular mortality

**Fig. 1:**
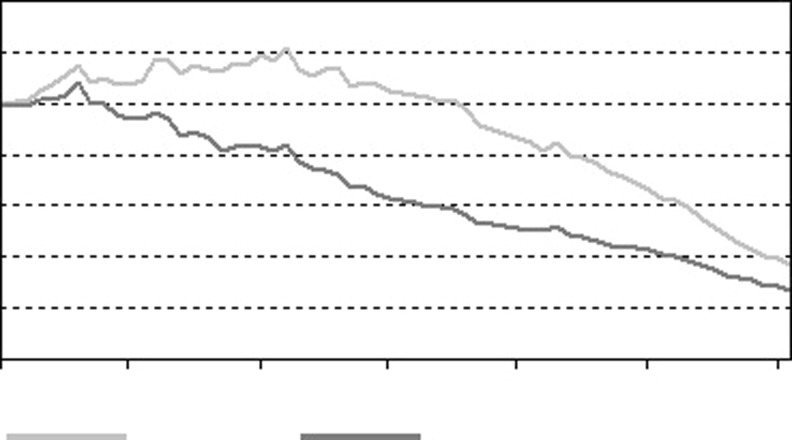
Standardised cardiovascular mortality per 100,000 inhabitants in the Netherlands in women and men from 1950 to 2011 [2]

Since its peak in the late 1950s (in women) and the early 1970s (in men), cardiovascular mortality in the Netherlands has gradually declined. This reduction has occurred despite the larger number of elderly persons as well as the more advanced age of contemporary Dutch citizens. After correction for these changes (standardisation), the drop in cardiovascular mortality that has taken place, a reduction of about 70 %, is considerable [1, 2]. Cardiovascular mortality from 1950 to 2012 is delineated in Fig. 1. After 1980, the cardiovascular mortality rates in women and men tend to converge [2].

Currently, CVD accounts for 27 % of all deaths. This percentage was 33 % 10 years ago, 37 % in 1992 and between 45 and 50 % in earlier decades. In the year 2012, 39,048 persons (20,733 women and 18,315 men) died from a primary cardiovascular cause (Table 1) [1]. The number of persons dying from CVD amounted to 46,942 in 2003, and thus a drop in absolute numbers of deaths from CVD has taken place concomitantly with the relative decrease in fatal CVD. Cardiovascular mortality as a fraction of total mortality in the last 10 years is presented in Table 1. The specific causes of cardiovascular mortality in the year 2012 are presented in Table 2 [1].

**Table 1:** Total and cardiovascular mortality in the Netherlands from 2003 to 2012 (Source: Statistics Netherlands (CBS))

Year	Cardiovascular mortality		Non-cardiovascular mortality		Total
	*N*	%	*N*	%	*N*
2003	46,942	33	94,994	67	141,936
2004	44,638	33	91,915	67	136,553
2005	43,350	32	93,052	68	136,402
2006	41,720	31	93,652	69	135,372
2007	40,849	31	92,173	69	133,022
2008	40,129	30	95,007	70	135,136
2009	38,897	29	95,338	71	134,235
2010	39,009	29	97,049	71	136,058
2011	38,132	28	97,609	72	135,741
2012	38,371	27	102,442	73	140,813

**Table 2:** Cardiovascular causes of death in the Netherlands in 2012 (Source: Statistics Netherlands (CBS)) [1]

Cause of death	Men		Women		Total	
	*N*	%	*N*	%	*N*	%
Ischaemic heart disease	5691	31	4029	19	9720	25
- Including myocardial infarction	3514		2681		6195	
Cerebrovascular disease	3302	18	5222	25	8524	22
Congenital heart disease	64	<1	46	<1	110	<1
Rheumatic and valvular heart disease	698	4	1023	5	1721	4
Infectious heart disease	331	2	435	2	766	2
Other heart diseases	5778	32	7543	36	13,321	34
- Including heart failure	2625		4136		6761	
- Including atrial fibrillation	538		939		1477	
Arterial vascular disease	1067	6	714	3	1781	5
Atherosclerosis and/or hypertension	1023	6	1227	6	2250	6
Other vascular disease	361	2	494	2	855	2
Total	18,315	100	20,733	100	39,048	100

Inspection of the data makes it clear that most cardiovascular deaths result from atherosclerotic disease affecting the coronary, cerebral, and other arterial vessels. Women die more often from more ‘mature’ atherosclerotic manifestations such as stroke and heart failure than men: the latter die in larger numbers from myocardial infarction (MI). However, one should appreciate that the primary cause of death is often difficult to ascertain and, in particular in persons over 80 years of age, may not be accurate [3, 4]. For instance, the number of 766 individuals supposedly dying from infectious CVD could well be false, since neither the incidence of bacterial endocarditis (estimated to be approximately 300 cases per year in the Netherlands) nor its clinical course (estimated case fatality less than 20 %) are able to account for the numbers reported [5]. Although the incidence of infective endocarditis has been stable in other parts of Europe over the past decades [6], it must be noted that contemporary data from the Netherlands are lacking.

On their own, atrial fibrillation (AF) and hypertension represent unlikely primary causes of death, yet thousands of deaths are attributed to these morbid conditions. Also, the mortality statistics combine causes and modes of death. For instance, heart failure is not a disease entity in itself, but merely a symptom of underlying cardiac conditions. Presumably many more persons die from the consequences of heart failure [7], but these deaths are attributed to for instance coronary, valvular, or congenital heart disease. This is likewise also the case for sudden cardiac death, which now only constitutes a minor part of the cause of death statistics (‘Other heart diseases’ in Table 2), whereas Dutch population-based studies have demonstrated that approximately 10 % of all deaths in adults fulfil the criteria for sudden cardiac death [8, 9].

## Coronary heart disease

**Fig. 2:**
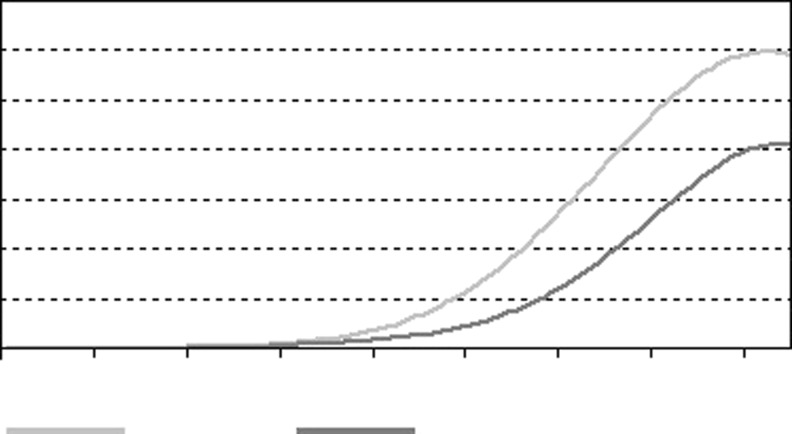
Coronary heart disease incidence in men and women (per 1000 inhabitants) in the Netherlands in 2007 (Source: General practice registry) [2]

On the basis of estimates from registries in the general population from 2007, 648,300 Dutch citizens had coronary heart disease (CHD) (estimated prevalence in women 3 %, and 5 % in men). Combined with the incidence of CHD in the same year (82,100 persons), the total number of women and men with CHD was estimated to be 730,400. Of these, an estimated 298,100 had angina [2]. Although men and women are struck by cerebrovascular disease at about the same age (data not shown), women develop CHD disease approximately 10 years later than their male counterparts (Fig. 2).

## Myocardial infarction

In 2012, 3514 men and 2681 women died from an MI (Tables 2 and 3).[1] Their absolute numbers in the years between 1980 and 2012 are presented in Table 3. MI-associated mortality declined significantly by more than 70 % over time in both men and women. Age-standardised mortality declined even further. The number of hospitalisations for MI in 2012 was 20,025 in men and 9653 in women. Between 1980 and 2012, the age-standardised MI admission rate (year of standardisation: 2012) declined by 42 % in men and by 23 % in women. However, the absolute number of hospitalisations for MI has not changed much [1].

**Table 3:** Number of fatal myocardial infarctions in the Netherlands from 1980 to 2012, unstandardised (Source: Statistics Netherlands (CBS)) [1]

Year	Men		Women	
	*N*	Per 100,000 inhabitants	*N*	Per 100,000 inhabitants
1980	12,634	180	7718	108
1985	12,486	174	8082	110
1990	10,002	135	7300	97
1995	8888	116	6800	87
2000	7291	93	5668	70
2005	5361	66	4141	50
2010	3840	47	2983	36
2012	3514	42	2681	32

Significant discordance in mortality statistics based on self-reports of hospitals (performance indicators) and data from clinical registries is noted. Hospital mortality from MI is between 5 and 10 % according to data from Statistics Netherlands (CBS), with age being the main determinant of adverse outcomes.

The incidence of first MI has been declining by about 3 to 4 % per year in the last decade, both in men and women. Total incidence declined by 38 % in men and by 32 % in women between 1998 and 2007 [10, 11].

## Surgical procedures

**Fig. 3:**
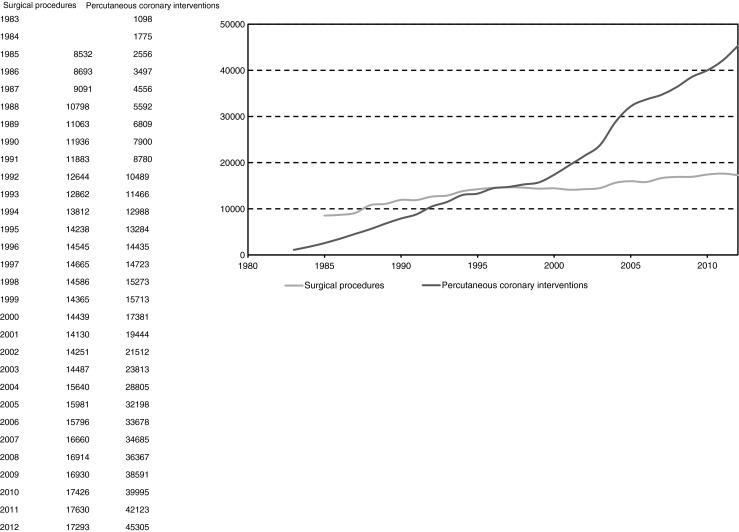
Number of surgical procedures and percutaneous coronary interventions in the Netherlands from 1983 to 2012 (Source: Supervisory Committee for Cardiac Interventions in the Netherlands (BHN)) [1]

The total number of surgical procedures, including paediatric surgery, has gradually increased over the years to 17,293 operations in 2012, performed in 16 surgical centres (Fig. 3) [1]. Seven percent of these interventions are ‘urgent’ (i.e. take place before the start of the next working day after the decision to operate has been taken) [12].

About two-thirds of the 16,262 operations in adults in 2012 involved coronary artery bypass surgery (CABG), while half of all surgical procedures are represented by isolated CABG (source: Supervisory Committee for Cardiac Interventions in the Netherlands (BHN)). In these procedures, the use of only arterial grafts has increased from 15 % in 1995 to nearly 26 % in 2011 [12]. The 30-day mortality of isolated CABG in 2010 was 1.2 % [1].

In 2012, a total of 3020 operations were related to diseases of the aortic valve, and in 41 % of these procedures coronary bypass grafts were implanted (source: Supervisory Committee for Cardiac Interventions in the Netherlands (BHN)). The type of prostheses implanted has gradually changed from mainly mechanical prostheses in the 1990s to the use of bio-prostheses in nearly 80 % of the operations nowadays. In addition to CABG and aortic valve surgery, mitral valve surgery is the most common indication for cardiac surgery with approximately 1750 operations annually. Concomitant CABG is performed in 36 % of these operations [1]. The proportion of mitral valve repairs has increased to over 75 %. Active endocarditis is reported in less than 200 surgical procedures per year [12]. Overall the 30-day mortality associated with valvular surgery in 2010 was 3.6 % [1].

Mainly as a result of varying availability of donor hearts, the number of yearly heart transplants has varied considerably over the years. On average, about 40 to 50 transplants are performed each year in three academic medical centres [13].

Other procedures include aortic surgery (approximately 1000 operations per year), transcatheter aortic valve implantation, and less frequently performed procedures such as surgical left ventricle reconstruction, ventricular septal rupture repair, rhythm surgery (as a concomitant or as a stand-alone procedure), surgery for correction of congenital heart disease, or management of cardiac trauma [14, 15].

## Percutaneous coronary interventions

While the number of surgical procedures has only risen gradually in the last decade, this has not been the case with the percutaneous interventions (PCIs). Their number has risen much more quickly in recent years. For instance, in 2012, 45,305 PCIs were performed in 30 centres, and this number represents more than a doubling of these procedures in a time frame of only 10 years (Fig. 3) [1, 16]. The most frequent indications for PCI were stable angina (42 %), acute MI (33 %) and unstable angina (22 %). Over 90 % of the procedures involved stent placement [13].

## ICD and pacemaker implants

There has been a substantial increase in the number of implantable cardioverter-defibrillator (ICD) implants in the last 10 years. Symptomatic patients with advanced heart failure (e.g. left ventricular ejection fraction <30 %) are the main recipients. Currently, their number is in the order of about 5000 new implants per year (Table 4). Registration of indications is not complete, but most (approximately 80 %) implants are prophylactic, i.e. implanted in patients at high risk for sudden cardiac death. The other patients receive an ICD after successful resuscitation. In addition to the new ICD implants, their replacement currently adds over 1000 procedures to the numbers presented in the table [13]. ICDs now have the ability to perform biventricular pacing in patients with heart failure and conduction abnormalities in order to emulate physiological cardiac function (i.e. cardiac resynchronisation therapy). Biventricular systems are becoming more popular and now account for almost 40 % of all procedures. The average age of the patients is 66 years and 23 % of them are women. Relatively few (6 %) ICDs are implanted beyond the age of 80 years.

**Table 4:** Number of new ICD implants based on 29 centres in the Netherlands from 2003 to 2012 (Source: Netherlands Heart Rhythm Association (NHRA)) [13]

Year	Non-CRT		CRT		Total
	*N*	%	*N*	%	*N*
2003	800	82	180	18	980
2004	1237	79	327	21	1564
2005	1724	72	665	28	2389
2006	2005	67	967	33	2972
2007	2590	72	1030	28	3620
2008	2644	69	1175	31	3819
2009	2890	68	1385	32	4275
2010	2986	62	1804	38	4790
2011	3192	61	2007	39	5199
2012	3051	63	1830	37	4881

Based on the Dutch ICD and Pacemaker Registry (DIPR), an additional 10,389 pacemakers (excluding cardiac resynchronisation therapy (CRT)) were implanted in 92 contributing hospitals in 2011. A fourth of the pacemaker implantations were replacements of another device.

## Heart failure

As with most other CVD, the presence of heart failure casts a shadow over the last phase of life in a considerable number of Dutch individuals. Between 20 and 30 % of the general population will develop some form of heart failure, usually when they are over 70 years of age [17]. Incidence rates in the Netherlands increase steeply from about 1 per 1000 person-years below 60 years of age to almost 50 per 1000 person-years in those aged 90 years and over.

Due to the ageing of the population, an increase in the number of patients with heart failure in the Netherlands was already predicted in the 1990s [18]. Contemporary data have confirmed that forecast, and an estimated 120,000 individuals with heart failure, about 1 % of the adult Dutch population, were reportedly present in 2008. The continuing ageing of the population is expected to raise their numbers to an approximate 200,000 in the coming decade [19].

The majority of heart failure is diagnosed in chronic stages, but episodes of acute cardiac decompensation can lead to hospitalisation. In 2011, 29,916 hospital admissions were registered in the Netherlands with heart failure as the primary discharge diagnosis (source: Dutch Hospital Data (LMR)) [11]. The numbers are equally distributed over both sexes. This number includes readmissions which are known to be frequent in patients with heart failure. Recent Dutch data on the ratio of first admissions to readmissions is lacking, but studies from U.S. registries consistently report 30-day readmission rates up to 25 % [20]. Between 1980 and 1990, the number of hospitalisations for heart failure increased by about 50 %. Thereafter, a decrease was observed but, as of 2002, the number of hospitalised patients increased again, although the total number of days spent in hospital has more or less stabilised [19].

The prognosis of patients with heart failure has been described as being more ‘malignant’ than that of many common cancers [21]. Five-year survival after the initial diagnosis ranges from approximately 25 to 35 % in population-based studies [17]. The in-hospital mortality associated with decompensated heart failure is poor, even for first occurrences, and has been reported to be in the order of 15 % in the Netherlands [17]. This is worse than the currently observed in-hospital mortality associated with acute MI.

**Fig. 4:**
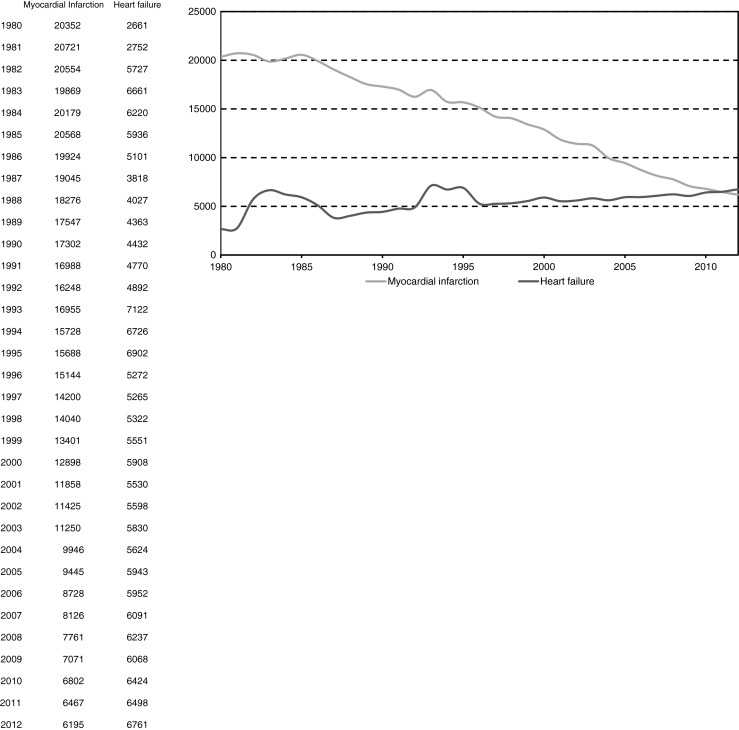
Number of deaths attributed to myocardial infarction or heart failure as the primary cause of death in the Netherlands from 1980 to 2012 (Source: Statistics Netherlands (CBS))

The absolute number of Dutch citizens dying either from MI or heart failure has been depicted in Fig. 4, and clearly shows the direction of the change in both causes of death. Since 2012, the number of deaths from heart failure (*n* = 6761) has surpassed the mortality from MI (*n* = 6195) (Table 2 and Fig. 4) [1].

## Atrial fibrillation

AF is the most common sustained cardiac arrhythmia. The condition carries serious health consequences, including increased risk for stroke and heart failure. AF is found to be present in about a quarter of patients presenting with an ischaemic stroke. The presence of many other cardio-metabolic disorders, such as coronary and valvular heart disease, cardiomyopathies, hypertension, and diabetes, predispose to the development of AF.

Estimates from a Dutch population-based cohort study indicate that the lifetime risk of AF is in the order of 20 to 25 %. As with heart failure, its occurrence strongly increases with age: the incidence rate of AF below 60 years of age is less than 1 per 1000 person-years, but rises to almost 20 per 1000 person-years in persons over 85 years of age [22]. These estimates may underestimate the true incidence since the presence of AF, either permanent or paroxysmal, may go undetected clinically.

On the basis of the best available data, an estimated 260,000 Dutch individuals are currently affected by AF [23]. These were responsible for 42,188 hospital admissions in 2011 with AF as the primary discharge diagnosis (source: Dutch Hospital Data (LMR)) [11]. Between 1994 and 2006, no major changes in its prevalence were observed but, given current demographic developments, an increase in the number of persons with AF in the coming years is projected [23].

## Hospitalisations for cardiovascular disease

During the last years, the number of hospitalisations associated with CVD has stabilised at around 400,000 (2400 per 100,000 inhabitants). Almost 115,000 (29 %) of these comprise 1 day admissions related to diagnostic or therapeutic procedures, or observations of cardiovascular symptoms. In the remaining cases, the mean duration of hospital stay is about 6 days (source: Statistics Netherlands (CBS)). Admission rates for rhythm disturbances are on the rise [11].

## Summary and conclusions

With plausible estimates of 730,000 patients with CHD, of 120,000 persons with heart failure, and of 260,000 men and women with AF, the total count of Dutch individuals with some manifestation of heart disease could be as high as one million. In addition, a substantial number of women and men have other forms of atherosclerotic disease, either of the cerebral and/or the peripheral arterial system. However, since atherosclerosis is usually not limited to one organ system and many cardiac and vascular diseases co-exist, the simple summation of the various disease manifestations will likely overestimate the number of individuals with cardiovascular and/or heart disease.

In view of the high CVD prevalence, it is striking that actual mortality from CVD has declined so much. Currently, 27 % of all deaths result from CVD, and cancer has overtaken CVD as the main cause of death in the Netherlands in both women and men (source: Statistics Netherlands (CBS)). Despite substantial risk factor burden [24, 25], cardiovascular mortality in the Netherlands is low in international comparisons. The current Dutch age-standardised mortality from circulatory disease is 147 per 100,000, and only Spain and France have lower cardiovascular mortality rates (143 and 126 per 100,000, respectively). In all other European countries, including for instance Switzerland and Greece, cardiovascular mortality is higher [26].

The number of cardiac procedures in the Netherlands, in particular that of PCI and CABG, is below the Organisation for Economic Cooperation and Development (OECD) average. As in most other European countries, the rate of PCI versus CABG is in the order of 2.5. Both the numbers of percutaneous and surgical cardiac procedures in the Netherlands are 50 % lower than in our affluent neighbouring countries, Belgium and Germany [26]. Patients with an acute MI are the main PCI target population with about 13,000 procedures in 30,000 patients hospitalised for MI. The 15,000 PCIs performed for stable angina constitute a relatively large proportion of PCI procedures, although the large number of patients with angina in the population must be taken into account.

Throughout our review we have cited various sources from which we obtained data on CVD in the Netherlands. However, data from different sources can be conflicting due to differential participation of hospitals in registries or due to the outcome definitions used. As an example, cardiovascular mortality is defined differently in the statistical updates from the Dutch Heart Foundation [10] compared to Statistics Netherlands (CBS). This results in minor discrepancies in the annual number of deaths attributable to CVD, in this case related to the inclusion or exclusion of congenital and perinatal heart disease and vascular autoimmune disorders (e.g. Table 1 and 2).

Future projections are always fraught with uncertainty, but demographic trends undeniably suggest an increase in the number of patients with heart disease and other forms of CVD in the not too distant future. Still, these trends are not dissimilar to the circumstances observed in the last decades, during which time cardiovascular mortality decreased so markedly. Most likely, these developments will continue, and high morbidity (and high prevalence) but relatively low mortality from CVD remains the most plausible scenario for the foreseeable future.

## Electronic supplementary material

Below is the link to the electronic supplementary material.

ESM 1 (PNG 5 kb)

ESM 2 (PNG 74 kb)
