# Erratum to: Heart disease in the Netherlands: a quantitative update

**DOI:** 10.1007/s12471-014-0532-1

**Published:** 2014-02-18

**Authors:** M. J. G. Leening, S. Siregar, I. Vaartjes, M. L. Bots, M. I. M. Versteegh, R.-J. M. van Geuns, J. J. Koolen, J. W. Deckers

**Affiliations:** 1 Department of Cardiology, Erasmus MC – University Medical Center Rotterdam, P.O. Box 2040, 3000 CA Rotterdam, the Netherlands; 2 Department of Epidemiology, Erasmus MC – University Medical Center Rotterdam, Rotterdam, the Netherlands; 3 Department of Cardiothoracic Surgery, Leiden University Medical Center, Leiden, the Netherlands; 4 Department of Epidemiology, Julius Center for Health Sciences and Primary Care, University Medical Center Utrecht, Utrecht, the Netherlands; 5 Dutch Heart Foundation, The Hague, the Netherlands; 6 Supervisory Committee for Cardiac Interventions in the Netherlands, Utrecht, the Netherlands; 7 Department of Cardiology, Catharina Hospital, Eindhoven, the Netherlands; 8 National Cardiovascular Data Registry, Amsterdam, the Netherlands

**Erratum to: Neth Heart J (2013) 22:3–10**

**DOI 10.1007/s12471-013-0504-x**

Figures 1, 2, 3, and 4 in the article were incorrect and should have appeared as presented in this erratum. The authors apologise for this oversight and any confusion this may have caused.

**Fig. 1:**
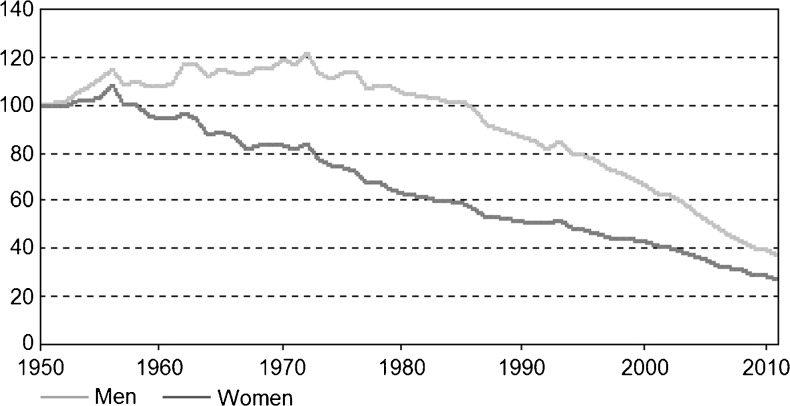
Standardised cardiovascular mortality per 100,000 inhabitants in the Netherlands from 1950 through 2011 [1]

**Fig. 2:**
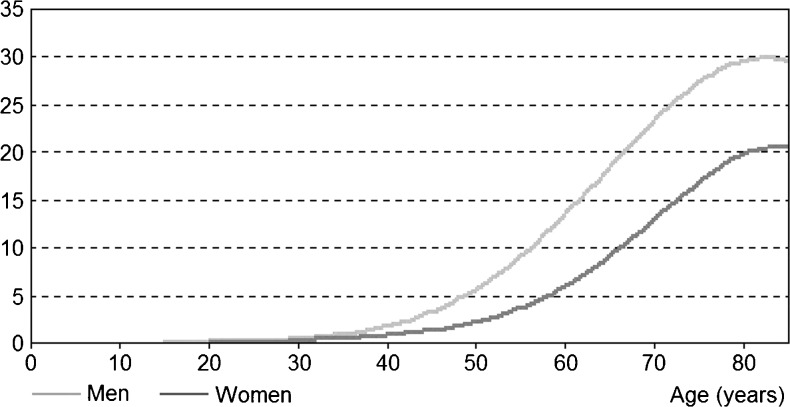
Coronary heart disease incidence per 1000 inhabitants in the Netherlands in 2007 (*Source*: general practice registry) [1]

**Fig. 3:**
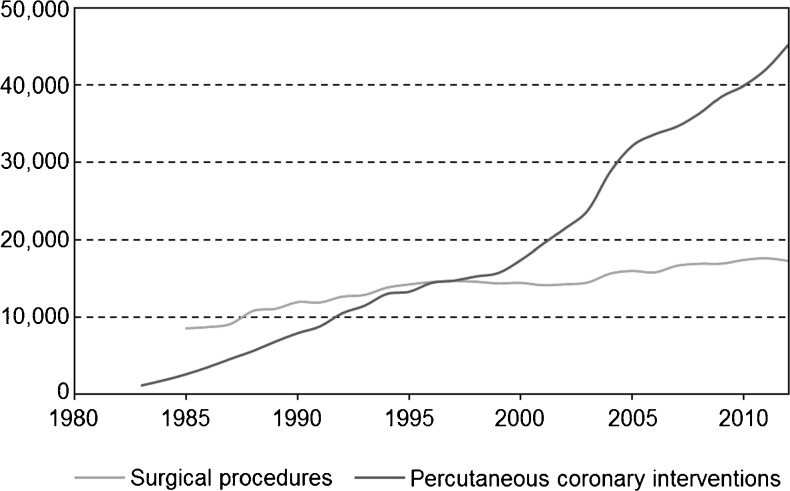
Number of surgical procedures and percutaneous coronary interventions in the Netherlands from 1983 through 2012 (*Source*: Supervisory Committee for Cardiac Interventions in the Netherlands (BHN)) [2]

**Fig. 4:**
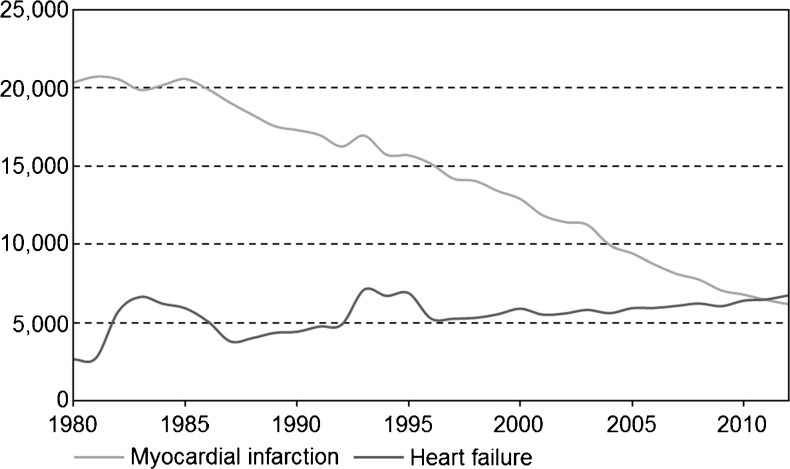
Number of deaths attributed to myocardial infarction or heart failure as the primary cause of death in the Netherlands from 1980 through 2012 (*Source*: Statistics Netherlands (CBS))
