# Supplementary figures and images for: Heart disease in the Netherlands: a quantitative update
Source: Neth Heart J. 2013 Dec 17;22(1):3–10. doi: 10.1007/s12471-013-0504-x (PMC3890010; doi:10.1007/s12471-013-0504-x)

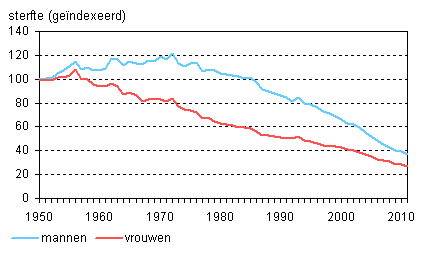

Supplement: Supplementary file 1 — (PNG 5 kb) [file 12471_2013_504_MOESM1_ESM.png]

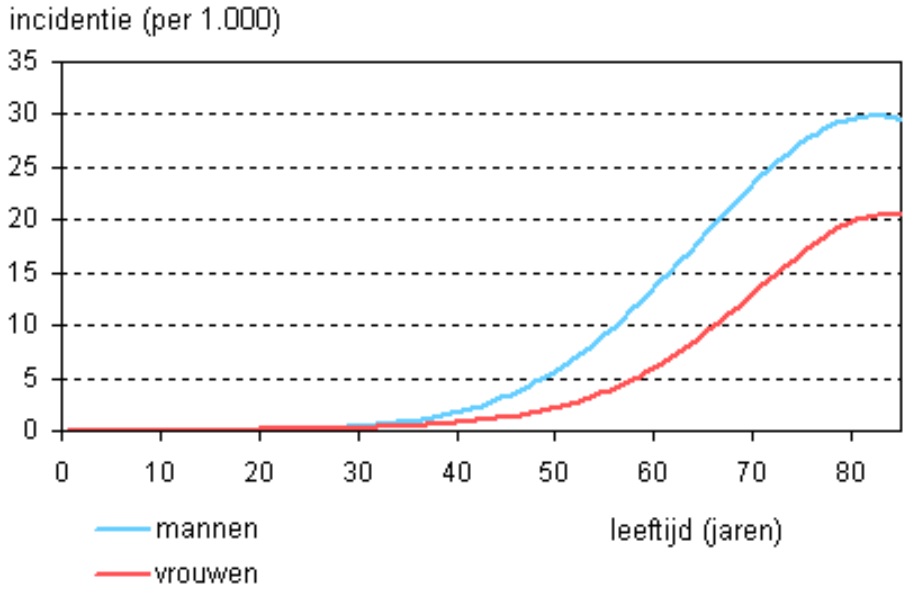

Supplement: Supplementary file 2 — (PNG 74 kb) [file 12471_2013_504_MOESM2_ESM.png]
